# First-Void Urine Microbiome in Women with *Chlamydia trachomatis* Infection

**DOI:** 10.3390/ijms23105625

**Published:** 2022-05-17

**Authors:** Valeria Gaspari, Camilla Ceccarani, Marco Severgnini, Gionathan Orioni, Tania Camboni, Luca Laghi, Sara Morselli, Claudio Foschi, Antonella Marangoni, Clarissa Consolandi, Bianca Maria Piraccini

**Affiliations:** 1 Department of Dermatology, IRCCS Azienda Ospedaliero-Universitaria di Bologna, 40138 Bologna, Italy; valeria.gaspari@aosp.bo.it (V.G.); gionathan.orioni@studio.unibo.it (G.O.); biancamaria.piraccini@unibo.it (B.M.P.); 2 Institute of Biomedical Technologies, National Research Council, 20054 Segrate, Italy; camilla.ceccarani@itb.cnr.it (C.C.); marco.severgnini@itb.cnr.it (M.S.); tania.camboni@itb.cnr.it (T.C.); 3 Department of Agricultural and Food Sciences, University of Bologna, 47521 Cesena, Italy; l.laghi@unibo.it; 4 Section of Microbiology, Department of Experimental, Diagnostic and Specialty Medicine, University of Bologna, 40138 Bologna, Italy; sara.morselli6@unibo.it (S.M.); antonella.marangoni@unibo.it (A.M.); 5 Microbiology Unit, IRCCS Azienda Ospedaliero-Universitaria di Bologna, 40138 Bologna, Italy

**Keywords:** *Chlamydia trachomatis*, urine, urobiome, microbiome, metabolome, urethritis

## Abstract

Background: *Chlamydia trachomatis* (CT) is the agent of the most common bacterial sexually transmitted infection worldwide. Until now, little information is available about the microbial composition of urine samples during CT urethritis. Therefore, in this study, we characterized the microbiome and metabolome profiles of first-void urines in a cohort of women with CT urethral infection attending an STI clinic. Methods: Based on CT positivity by nucleic acid amplification techniques on urine samples, the enrolled women were divided into two groups, i.e., “CT-negative” (*n* = 21) and “CT-positive” (*n* = 11). Urine samples were employed for (i) the microbiome profile analysis by means of 16s rRNA gene sequencing and (ii) the metabolome analysis by ^1^H-NMR. Results: Irrespective of CT infection, the microbiome of first-void urines was mainly dominated by *Lactobacillus*, *L. iners* and *L. crispatus* being the most represented species. CT-positive samples were characterized by reduced microbial biodiversity compared to the controls. Moreover, a significant reduction of the Mycoplasmataceae family—in particular, of the *Ureaplasma parvum* species—was observed during CT infection. The *Chlamydia* genus was positively correlated with urine hippurate and lactulose. Conclusions: These data can help elucidate the pathogenesis of chlamydial urogenital infections, as well as to set up innovative diagnostic and therapeutic approaches.

## 1. Introduction

*Chlamydia trachomatis* (CT) is the agent of the most common bacterial sexually transmitted infection (STI) worldwide, with a relevant clinic and economic impact [1]. In women, urogenital CT infections (i.e., urethritis and cervicitis) are often asymptomatic and, if left untreated, can lead to several complications and sequelae, such as pelvic inflammatory disease, tubal infertility, and ectopic pregnancy [2,3]. Moreover, urogenital infections are associated with an increased likelihood of HIV infection transmission and acquisition [2].

CT is an obligate intracellular pathogen with a cycle of development characterized by two distinct bacterial forms. The elementary bodies (EBs), infectious but non-dividing, enter the epithelial cells and differentiate into reticulate bodies (RBs). After several rounds of replication, the RBs differentiate back into EBs and are released from the host cell 48–72 h post-infection, ready to infect neighboring cells [1,2,3].

During the infection, CT interacts with the endogenous and commensal microorganisms of the urogenital tract, competing for nutrients and creating peculiar microbial and metabolic interactions with the host [4,5].

In this context, recently, many authors have focused on the correlation between CT infection and the vaginal environment [6,7,8,9,10,11]. Some cross-sectional studies have demonstrated that the presence of bacterial vaginosis (BV), a dysbiosis status with a depletion of lactobacilli and the predominance of other anaerobic species, increases the risk of STI acquisition, including genital CT infections [6,7,8]. Indeed, the vaginal environment of CT-infected women is usually characterized by a decrease in *Lactobacillus* spp., together with a significant increase in dysbiosis-associated bacterial taxa, such as *Megasphaera* spp., *Atopobium vaginae*, *Gardnerella vaginalis*, and *Prevotella* spp., creating a highly complex, polymicrobial community [12,13,14]. A network of different anaerobes, such as *Gardnerella vaginalis*, *Prevotella amnii*, *Prevotella buccalis*, *Prevotella timonensis*, *Aerococcus christensenii*, and *Variovorax guangxiensis*, has been identified as a potential biomarker of CT genital infection [11]. Moreover, in CT-positive patients, the vaginal microbiota is often dominated by *Lactobacillus iners*, a transitional species characterized by low antimicrobial activity [9].

These microbial signatures are usually accompanied by modifications in the metabolic profiles of the vaginal niche, such as the reduction in some amino acids (e.g., tyrosine and glutamate) and the trend towards the increase of some biogenic amines (putrescine, cadaverine, and trimethylamine) and short chain fatty acids (especially acetate and succinate) [13].

Although the characteristics of the vaginal microbiome during chlamydial infections have been largely investigated, little information is available about the microbial/metabolic compositions of urine samples in the case of CT urethritis [4].

Therefore, the aim of this study was to characterize the microbiome and metabolome profiles of first-void urines in a cohort of women with CT urethral infection. In particular, by means of 16S rRNA gene sequencing and proton-based nuclear magnetic resonance (^1^H-NMR) spectroscopy, we studied the microbial and metabolic compositions of urine samples in CT-positive and CT-negative young women attending a STI clinic.

## 2. Results

### 2.1. Study Population

Initially, our dataset consisted of a total of 40 women (15 CT-positive and 25 CT-negative) meeting the inclusion criteria; eight samples (four CT-positive and four CT-negative) were subsequently excluded due to a low quantity of raw sequencing reads (i.e., <5000). Therefore, the final dataset consisted of 32 women divided into 2 groups of patients: a CT-negative group (no CT infection, *n* = 21) and patients with CT infection (CT positivity, *n* = 11).

No significant difference in the mean ages between the groups was noticed (CT-negative: 27.3 ± 6.9 years; CT-positive: 24.3 ± 3.4 years; *p* = 0.19). Notably, most (7/11; 63.6%) of the CT-positive patients denied the presence of urogenital symptoms. Dyspareunia (three cases) and vaginal spotting (two cases) were the most reported disorders. Only one woman complained about the presence of vaginal discharge and another one about urinary/urethral symptoms (i.e., dysuria).

### 2.2. CT Genotyping

The most common CT serovar in our population was E (5/11; 45.4%), followed by F (3/11; 27.2%), G (2/11; 18.2%), and D (1/11; 9.1%). No case due to the L1–L3 serovars (responsible for lymphogranuloma venereum) was found.

### 2.3. Taxonomic Composition of Urine Bacterial Communities

**Figure 1:**
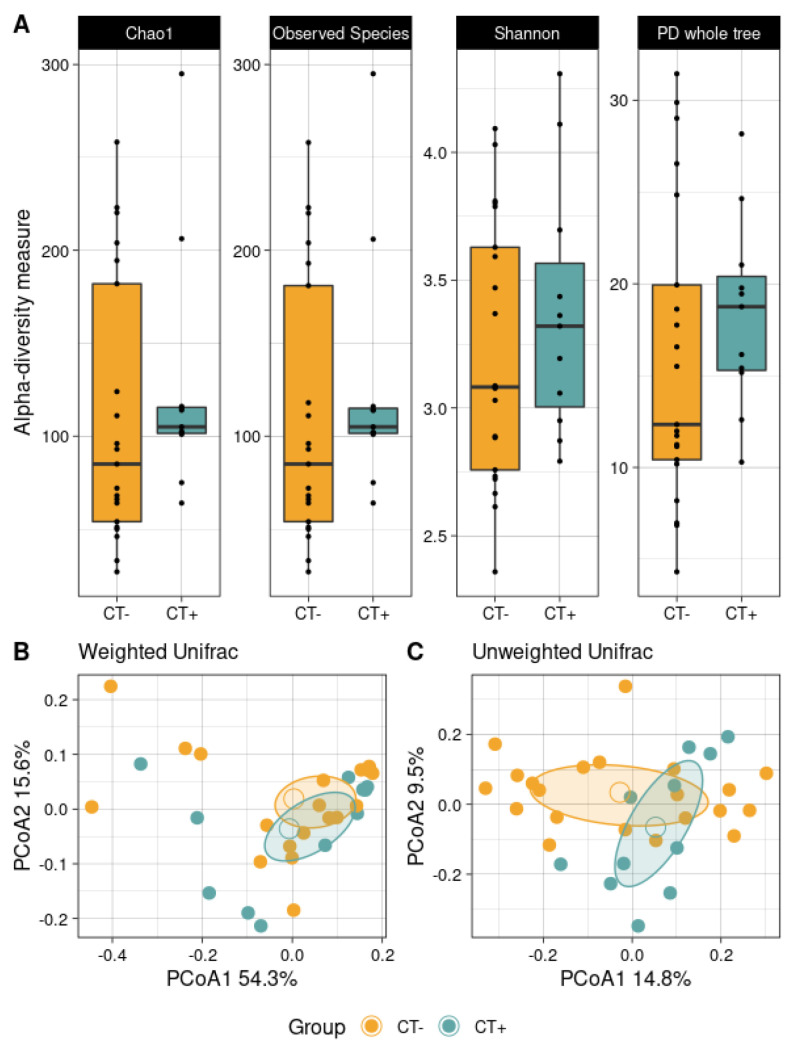
Alpha and beta diversity. (**A**) Boxplots of the infection status (not-infected patients, CT−; CT-infected patients, CT+) showing the biodiversity distribution according to four metrics, accounting for both species richness and evenness. (**B**) Principal coordinates analysis (PCoA) of the beta-diversity weighted and (**C**) unweighted Unifrac distance matrix. The unweighted distance was found significantly different between infection groups (*p*-value = 0.0254). First and second principal component axes are reported; plots also show an average centroid and confidence ellipse.

Bacterial biodiversity was found reduced in samples with CT infection, although not significantly for all the metrics taken into consideration, for both species richness and biodiversity (Chao1, *p* = 0.184; Observed Species, *p* = 0.183; Shannon index, *p* = 0.435; and Faith’s Phylogenetic Distance, *p* = 0.254) due to some outlier samples (Figure 1A). Beta-diversity, instead, revealed a significant difference in CT-negative and CT-positive sample distribution within the unweighted Unifrac distance matrix (*p* = 0.0254; weighted, *p* = 0.446) (Figure 1B,C).

**Figure 2:**
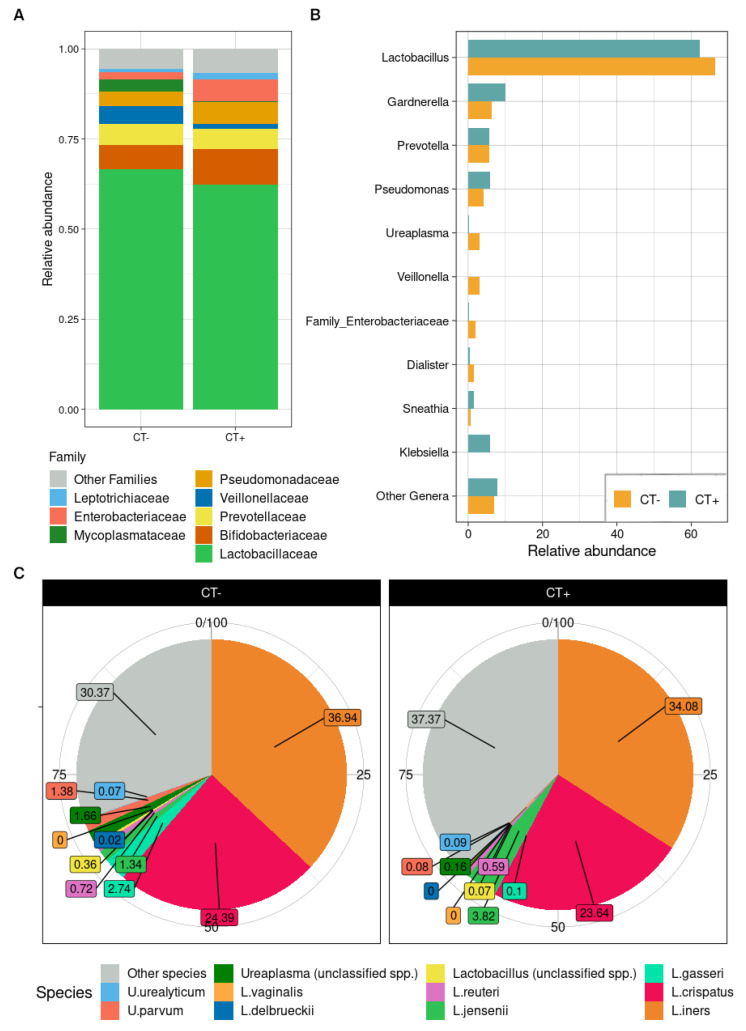
Taxonomy characterization. (**A**) Stacked bar plot at the family level of the groups (not-infected patients, CT−; CT-infected patients, CT+). (**B**) Dodged bar plot at the genus level. For both plots, “Other Families/Other Genera” lists all the bacterial groups with their relative abundance < 1% in at least one of the patient groups. (**C**) Pie chart of the relative abundance of the genera *Ureaplasma* and *Lactobacillus* at the species level; “unclassified” represent all the species level reads not uniquely assigned to any of the species available in the NCBI database for the two genera analyzed. “Other species” comprehends all the other genera but those two.

The relative abundances of the two experimental groups did not differ in a clear and drastic way, being quite similar among patients with and without CT infection. CT-negative patients were found to have a higher proportion of the Firmicutes phylum, whereas CT-positives were more enriched in Actinobacteria and Proteobacteria. At the family level, CT-infected women had increased abundances of *Bifidobacteriaceae* and *Enterobacteriaceae*, with fewer amounts of *Lactobacillaceae* and *Veillonellaceae* (Figure 2A). The only significant difference among the experimental groups, though, was with the *Mycoplasmataceae* family consistently reduced in CT-positive patients (average abundance: 3.24% in CT-negative vs. 0.37% in CT-positive, *p* = 0.014). To this last family belongs the only bacterial genus found statistically different between the infection groups: *Ureaplasma* (3.10% in CT-negative vs. 0.33% in CT-positive, *p* = 0.013). Few differences observed within the CT-positive samples at the genus level comprised an increase in *Gardnerella*, *Pseudomonas*, *Klebsiella*, and *Chlamydia* spp. and a reduction in *Lactobacillus* and *Veillonella* (Figure 2B). Some of the incremented genera were also found highly positively correlated in the co-abundance analysis (Figure S1) and, thus, formed a co-abundant group (CAG) comprising *Dialister*, *Prevotella*, *Atopobium*, *Gardnerella*, *Megasphaera*, *Sneathia*, and *Fusobacterium*; we named this CAG after the most abundant genus as “*Gardnerella*-CAG”. *Chlamydia* and *Lactobacillus* together formed another CAG characterized by negative co-abundance relationships with *Gardnerella*-CAG; *Streptococcus*, *Veillonella*, and *Pseudomonas* were members of another CAG, as *Klebsiella* and unclassified genera of the Enterobacteriaceae family comprised another co-abundant group; *Ureaplasma* did not group together with other genera but showed positive relationships with *Gardnerella*-CAG. Considering each CAG as the sum of the bacterial genera relative abundance, no significant difference was observed between the groups, apart from *Ureaplasma*, as it was ungrouped to other bacteria and replicated the previously described result (*p* = 0.013).

Overall, the dominant bacterial genus for the urobiota of our entire dataset was *Lactobacillus* (accounting for 66.52% in the CT-negative samples and 62.30% in the CT-positive ones); given this predominance and the significant reduction of *Ureaplasma* in CT-infected patients, we performed a focused species analysis on these two genera. *L. jensenii* was mainly found in CT-positive patients (3.82% vs. 1.34% in the CT-negative samples), whereas *L. gasseri* was only present in the CT-negative ones (2.74% vs. 0.01% in the CT-positive). *L. iners* and *L. crispatus*, instead, shared a comparable relative abundance within the two groups (respectively, 36.94% and 24.39% in the CT-negative vs. 34.08% and 23.64% in the CT-positive). As for the *Ureaplasma* genus, we observed a significant reduction of *U. parvum* in the CT-infected samples (0.08% compared to 1.38% in the CT-negative; *p* = 0.013), while *U. urealyticum* did not change with chlamydial infection (Figure 2C). All taxonomic findings are displayed in Table S1.

### 2.4. Urine Metabolites Composition and Metabolite Microbiome Correlation

By means of the metabolomic analysis, we detected and quantified 125 candidate substances, mainly belonging to the groups of sugars, organic acids, nitrogen compounds, and amino acids.

**Figure 3:**
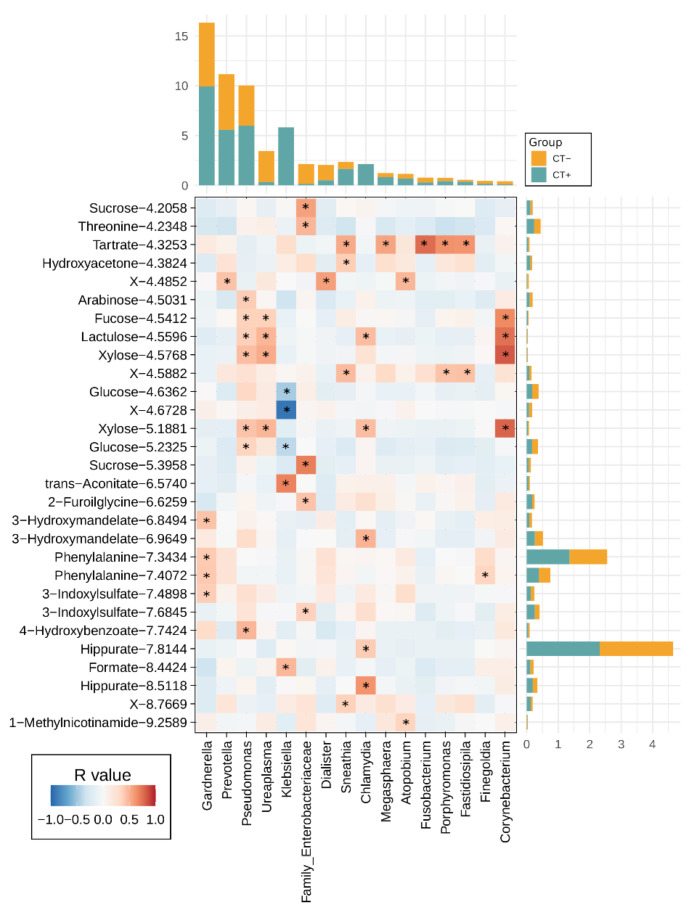
Correlation between the genus abundances and metabolic compounds. Heatmap reporting the positive (red) and negative (blue) Pearson correlations between bacterial groups (at the genus level, with corresponding relative abundances stacked on top, for both patient groups) and the metabolites identified through the ^1^H-NMR analysis (abundances stacked on the right). Asterisks indicate statistically significant correlations (asymptotic *p*-value < 0.05). Only genera and metabolites with at least one significant correlation are reported in the heatmap, i.e., 16 genera and 29 metabolites.

A correlation between all the urine metabolites quantified by ^1^H-NMR and the 19 most abundant bacterial genera was performed (Figure 3). The *Chlamydia* genus was positively correlated with hippurate metabolite (“Hippurate 8.5118”, R = 0.625 and “Hippurate 7.8144”, R = 0.395) and with lactulose (R = 0.4566). Lactulose showed a positive relationship with *Ureaplasma*, as well (R = 0.487). In addition, this latter genus was found positively correlated with the sugar xylose (“Xylose 5.1881”, R = 0.484 and “Xylose 4.5768”, R = 0.529) and with the sugar fucose (R = 0.372). Of all the main genera analyzed, *Klebsiella* was the only bacterial group that reported negative correlations—in particular, with the sugar glucose (“Glucose 5.2325”, R = −0.396 and “Glucose 4.6362”, R = −0.477).

## 3. Discussion

The knowledge about the composition of the microbiome and metabolome of first-void urine samples during CT infection can help elucidate the pathogenesis of chlamydial urogenital infections, as well as to set up new diagnostic tools (e.g., detection of specific metabolites) and innovative therapeutic approaches (e.g., probiotic supplementation). Thus, in this study, we characterized the microbial and metabolic profiles of first-void urines in a cohort of women with CT urethral infection.

As a strength of our work, to avoid biases in the results, we excluded from the study several conditions interfering with the urogenital environment, such as the recent use of any drugs or medications and the presence of chronic diseases and major endocrine or gynecologic pathologies. Moreover, we demonstrated that chlamydial infections were due to “non-invasive” CT-serovars (D-K), excluding the presence of cases of lymphogranuloma venereum, potentially responsible for significant and profound changes of the urogenital microbiome.

At first, irrespective of the presence of chlamydial infection, we observed that the microbiome of first-void urines was mainly dominated by the *Lactobacillus* genus, with *L. iners* and *L. crispatus* as its most represented species. These data agree with recent observations, showing that the most abundant genus found in the urinary microbiome in women is *Lactobacillus*, *L. crispatus* as the hallmark of a state of health [15,16]. Considering that a close relationship exists between vaginal and urinary microbiota [17], it was not surprising to find a high proportion of *L. iners* in urines. Indeed, this species has been reported to be the dominating taxon in a large subset of women worldwide, its presence associated with young age and unprotected sexual practices [18,19].

It should be noted that the urine microbiome of a subset of women was enriched in bacterial genera other than *Lactobacillus*, such as *Gardnerella*, *Prevotella*, *Dialister*, and *Atopobium*. These results reflect the existence of different urinary bacterial communities grouped into urotypes in which a particular bacterial genus predominates [20]. As previously shown, urine specimens can be classified into urotypes defined by the predominant (>50% abundance) bacterial taxon present. In line with our findings, the most common urotype in continent adult women is *Lactobacillus*, followed by *Streptococcus* and *Gardnerella* [21]. Urotypes dominated by *Lactobacillus* and *Gardnerella* are often found in younger women, and the latter can also be found in subjects without evidence of any a clinical disorder [21].

This aspect contrasts with what happens in the vaginal tract, where the predominance of *Gardnerella* and other anaerobic bacteria is associated with conditions of dysbiosis (i.e., bacterial vaginosis), often with the presence of genital signs and symptoms [13].

Interesting data emerged when considering CT-infected women. CT-positive samples were characterized by reduced microbial biodiversity, indicating the ability of chlamydia to take over the bacterial inhabitants of the microbiome, probably by means of different mechanisms, including a competition for nutrients and adhesion sites, and modulation of the host immune response [5]. However, the urinary microbiome composition of the CT-positive samples was quite similar to the microbial profiles of the CT-negative groups. Only a significant reduction of the Mycoplasmataceae family—in particular, of the *Ureaplasma parvum* species—was observed during CT infection.

Considering the intracellular localization of chlamydial infections and the high stability of the urinary microbiome, is it likely that CT induces only slight modifications of the bacterial residents of the urethral tract [22,23].

The significantly lower levels of *Ureaplasma parvum* found in the CT-positive samples could reflect a bacterial competition for cellular adhesion. *Ureaplasma parvum* is a member of the class of Mollicutes, frequently found in the urogenital ecosystem of asymptomatic “healthy” women as a commensal colonizer [24]. Mycoplasmas adhere to epithelial cells by means of individual surface-associated adhesins, such as multiple-banded antigens, and possesses the ability to form a biofilm [25,26]. Thus, we can speculate that a higher abundance of *U. parvum* in the urogenital tract could be a “protective factor”, preventing the subsequent adhesion and colonization of exogenous pathogens, including chlamydia.

Our findings are in line with what was previously observed for the vaginal microbiome, where higher *U. parvum* bacterial loads were detected in “healthy” women compared to CT-positive subjects [24].

Nevertheless, we cannot rule out that the significant reduction of *U. parvum* in CT-infected women follows the onset of chlamydial infection as a consequence of CT replication and/or activation of the immune and inflammatory response.

Therefore, further studies are needed to assess if the changes in the *U. parvum* levels precede or follow CT infection. In addition, it will be necessary to understand if *U. parvum* is a real “beneficial microbe” or a simple bystander of the urogenital tract, deciphering the molecular mechanisms of the interactions between mycoplasmas, chlamydia, and the host.

Even though not statistically significant, a different distribution of some *Lactobacillus* species was observed among the experimental groups. Indeed, *L. jensenii* was mainly found in CT-positive patients, whereas *L. gasseri* was only present in CT-negative ones. In this context, it should be remembered that each *Lactobacillus* species exerts a different antimicrobial activity, related to a different ability to produce bioactive substances and to compete for adhesion and nutrients [27]. As well as *L. crispatus*, *L. gasseri* is often found in the genital microbiota of healthy premenopausal women, associated with a condition of eubiosis [28]. Thus, its presence can help protect the urogenital environment from exogenous pathogens, such as CT.

CT-positive women were also characterized by a higher abundance, although not a significant one, of *Klebsiella* spp. and *Gardnerella* spp. In the vaginal tract, these species are commonly found during dysbiotic conditions, (i.e., aerobic vaginitis and bacterial vaginosis, respectively), thus increasing the likelihood of exogenous infections [13,29]. Thus, the presence of *Klebsiella* spp. and *Gardnerella* in the urogenital tract can have a similar significance/effect, associated with a lower protection against pathogens, including CT.

When urine metabolite concentrations were related to the bacterial composition, we highlighted peculiar microbial/metabolic fingerprints. Among them, we found a significantly positive correlation between the *Chlamydia* genus and hippurate.

Modifications of hippurate concentrations in urine samples have been previously associated with several conditions, including metabolic disorders and systemic infectious diseases [30,31,32,33]. As an example, patients with tuberculosis infection are characterized by a lower concentration of urine hippurate, probably due to the synthesis of aromatic amino acids such as tryptophan, tyrosine, and phenylalanine [32].

The intracellular growth and pathogenesis of *Chlamydia* species are controlled by the availability of the aromatic amino acid tryptophan [34]. In particular, CT is a tryptophan auxotroph and cannot synthesize tryptophan de novo but only via indole salvage [35]. Probably, the increased levels of urine hippurate could be attributed to the peculiar metabolic needs of CT, involving the metabolism of amino acids and other nitrogen sources [13]. Nevertheless, further studies are needed to better clarify the origin of urine hippurate and to deepen the metabolic pathways used by CT to adapt to the urethral microenvironment.

Moreover, we found a positive correlation between the *Ureaplasma* genus and several sugars. This aspect should be taken into account if we consider that sugars can have several effects on CT survival and virulence. For example, it has been recently shown that the depletion of glucose is associated with a significant reduction in CT elementary body infectivity [36]. Additionally, it has been found that the concentrations of certain sugars (i.e., sucrose and mannitol) in the urethral lumen could favor CT acquisition or could be of aid in bacterial viability [37]. Thus, the action of members of the *Ureaplasma* genus on sugar/carbohydrate metabolism could represent another way of protection displayed by mycoplasmas against CT. Indeed, these microorganisms can compete for nutritional substrates, interfering with the sugar availability in the urogenital environment.

We are fully aware of some limitations of the study. At first, the lack of information about the presence and number of leukocytes in the urethral fluids/urines made any association between the microbial/metabolic changes and inflammation impossible. Second, the availability of data about the vaginal microbiome/metabolome would have helped in understanding the dynamics between these two interconnected ecological niches. 

Even though our data are mainly preliminary and descriptive, this study can open the way to the use of new “omic” sciences to deepen host–pathogen interactions and to better understand CT pathogenesis.

For the first time, we assessed the urine microbiome in women with CT urogenital infection. Further studies, including a larger cohort of women, are needed to understand the accurate origin of the urine metabolites and to comprehend if the observed alterations precede or follow the onset of infection. Moreover, the potential role of the microbial and metabolic changes in the pathogenesis of CT infection, as well as their diagnostic/prognostic use, should be investigated.

## 4. Materials and Methods

### 4.1. Study Population and Sample Collection

From May to July 2016, all the premenopausal nonpregnant Caucasian women attending the STI Outpatients Clinic of Sant’Orsola-Malpighi Hospital in Bologna (Italy) and presenting risk factors for CT infection (age < 25 years, new or multiple sexual partners, and unsafe intercourses) were enrolled. Exclusion criteria included age under 18; the use of any antimicrobial, probiotic, or contraceptive in the month preceding the study; the presence of chronic diseases and major endocrine or gynecologic pathologies; and the positivity for any microbial agent responsible for urethritis other than CT (e.g., *Neisseria gonorrhoeae* or *Mycoplasma genitalium*).

For all the patients, demographic data and information about their urogenital symptoms were recorded. Written consent was obtained by all the patients, and the study protocol was reviewed and approved by the Ethics Committee of St. Orsola-Malpighi Hospital (7/2016/U/Tess).

Each enrolled woman provided the first void of the first urine of the morning. Then, after the interview and a clinical examination, a vaginal swab (E-swab, Copan, Brescia, Italy) was collected by a clinician. The urine specimens were split within 3 h of collection: 2.5 mL were used for CT, *Neisseria gonorrhoeae*, *Trichomonas vaginalis*, and *Mycoplasma genitalium* detection by commercial nucleic acid amplification techniques (NAATs) (Versant CT/GC DNA 1.0 Assay; Siemens Healthineers, Terrytown, NY, USA; Aptima *Trichomonas vaginalis* and Aptima *Mycoplasma genitalium* assay, Panther system, Hologic, Marlborough, MA, USA, respectively), whereas 1 mL was frozen at −20 °C and thawed only at the time of the metabolomic analysis, as described below.

The vaginal swabs were used for the molecular detection of CT, *N. gonorrhoeae*, *T. vaginalis*, and *M. genitalium*, as described for urine samples.

Eligible women were allocated to one of the two following groups according to the CT positivity (CT-positive group) or negativity (CT-negative group) in both the urogenital-tested sites (i.e., vaginal swab and urine).

### 4.2. CT Genotyping

CT-positive samples underwent a molecular genotyping, starting from the correspondent remaining eluate of the Versant PCR plate [38]. Molecular genotyping was performed by *omp1* gene semi-nested PCR, followed by a RFLP analysis, as previously described [3]. Briefly, the first product of 1033 base pairs (bp) was amplified using the following paired primers: SERO1A (5′-ATGAAAAAACTCTGAAATCGG-3′) and SERO2A (5′-TTTCTAGATCTTCATTCTTGTT-3′). Then, 1 μL of the first-round PCR product was used to amplify a 978-bp fragment using the following primers: SERO2A and PCTM3 (5′-TCCTTGCAAGCTCTGCCTGTGGGGAATCCT-3′). After the PCR step, the amplified product was digested with *AluI*, *DdeI*, and/or *HinfI* as restriction enzymes (Promega, Madison, WI, USA) and visualized after the electrophoresis run in ethidium bromide stained 12% polyacrylamide gel. CT serovar identification was achieved by the analysis of the specific restriction pattern.

### 4.3. Urine Microbiome Analysis

Urine microbiome profiles were analyzed starting from the remaining DNA eluate of the Versant PCR plate [38]. The V3–V4 hypervariable regions of the bacterial 16S rRNA gene were amplified according to the 16S metagenomic sequencing library preparation protocol (Illumina, San Diego, CA, USA). The final indexed libraries were pooled at 6 pmol/L for a 2 × 300-bp paired-end run on the Illumina MiSeq platform.

Amplicon sequence variants (ASVs) were identified from 16S paired-end sequencing using the Divisive Amplicon Denoising Algorithm (DADA2) pipeline, including filtering and trimming of the reads (version 1.16.0) [39]. Reads per sample were trimmed to 5000 reads in order to compensate for the sequencing unevenness of the samples and to provide a consistent minimum amount for the downstream analysis, carried out through the “phyloseq” package (version 1.34.0) [40]. Alpha-diversity evaluation was performed according to several microbial diversity metrics (i.e., chao1, Shannon Index, observed species, and Faith’s phylogenetic distance). Beta-diversity analysis was conducted using both weighted and unweighted Unifrac metrics [41] and through the principal coordinates analysis (PCoA).

Taxonomy was assigned to the ASVs using the 8-mer-based classifier from the 11.5 release of the RDP database and using the SILVA 16S rRNA database (release 138) [42,43]. Characterization of *Lactobacillus* and of *Ureaplasma* spp. were performed by BLAST-aligning all ASVs belonging to those bacterial genera to two custom reference databases made up from collecting all available reference sequences in the NIH-NCBI database (ftp://ftp.ncbi.nlm.nih.gov/genomes/GENOME_REPORTS/prokaryotes.txt, accessed on 15 February 2022) of the 17 main *Lactobacillus* species commonly found in the vaginal environment and of the 2 annotated *Ureaplasma* species (i.e., *U. parvum* and *U. urealyticum*) for both genera, selecting only sequences with a completion status of the “complete genome”, “chromosome”, or “scaffold”. Potential matches were filtered to retrieve an unequivocal classification for each read, according to the procedures already described [13].

### 4.4. Urine Metabolome Analysis by ^1^H-NMR

Urine samples were prepared for ^1^H-NMR analysis by thawing them right before analysis. After centrifugation for 15 min at 18,630× *g* at 4 °C, an amount of supernatant equal to 700 μL was added to 200 μL of a D_2_O solution of 3-(trimethylsilyl)-propionic-2,2,3,3-d4 acid (TSP) sodium salt 10 mM buffered at pH 7.00 ± 0.02 by means of 1M phosphate buffer. ^1^H-NMR spectra were recorded at 298 K with an AVANCE III spectrometer (Bruker, Milan, Italy) operating at a frequency of 600.13 MHz and equipped with Topspin software (Ver. 3.5) [44].

To each spectrum, line broadening (0.3 Hz) and phase adjustment were applied by Topspin software, while any further spectra processing, molecules quantification, and the data mining step were performed in R computational language (R: A Language and Environment for Statistical Computing, v. 4.0.5) by means of scripts developed in house.

The spectra were aligned towards the right peak of the alanine doublet, set to 1.473 ppm, and the signals of water and urea were removed. The spectra were then baseline-adjusted by means of peak detection according to the “rolling ball” principle implemented in the “baseline” R package [45,46].

The signals were assigned by comparing their chemical shift and multiplicity with the Human Metabolome Database [47] and the compound library (Ver. 10) of Chenomx software (Chenomx Inc., Edmonton, AB, Canada, Ver. 8.3). Quantification of the molecules was performed in the first sample, acquired by employing the added TSP as an internal standard. To compensate for differences in the solids content, any other sample was then normalized to such a sample by means of probabilistic quotient normalization [48]. Integration of the signals was performed for each molecule by means of rectangular integration.

### 4.5. Data Analysis and Statistics

Statistical evaluation of the beta-diversity differences was assessed by a permutation test with pseudo-F-ratios using the “adonis” function from R package “vegan” (version 2.0-10) [49], while alpha-diversity and pairwise taxonomy abundance analyses were performed using nonparametric Mann–Whitney *U* tests.

Co-abundance analysis (CAG) was performed using the Euclidean correlation with Ward clustering on a matrix of the 15 most abundant bacterial groups at the genus level. Metabolite concentrations were correlated to the bacterial composition by calculating the Spearman’s correlation coefficient between metabolites and bacterial genera present ≥ 1% in at least one sample. For each statistical analysis, *p*-values < 0.05 were considered as significant. All statistical analyses were performed using the R software (version 4.0.2).

## Data Availability

The raw sequencing data are available in the National Center for Biotechnology Information (NCBI) Sequence Read Archive (SRA) under the BioProject accession number PRJNA812368 (https://www.ncbi.nlm.nih.gov/sra/; accessed on 15 February 2022). Raw metabolomic data are available in the Supplementary Materials (Data Sheet S1).

## Supplementary Materials

The following supporting information can be downloaded at: https://www.mdpi.com/article/10.3390/ijms23105625/s1.
