# Supplementary material for: First-Void Urine Microbiome in Women with Chlamydia trachomatis Infection
Source: Int J Mol Sci. 2022 May 17;23(10):5625. doi: 10.3390/ijms23105625 (PMC9143427; doi:10.3390/ijms23105625)
Supplement: Supplementary file 1 [file ijms-23-05625-s001.zip › Supplementary materials.pdf]

Supplementary materials

Figure S1. Co-abundance analysis and clustering. Heatmap reporting the co-presence (red) and co-absence (blue) Pearson correlation between the 15 most abundant bacterial genera. Bacteria have been clustered for co-presence similarity into 5 Co-Abundant Groups (CAG) through Ward linkage, and CAGs have been named after the most abundant genus: "*Gardnerella*-CAG" (yellow), "*Ureaplasma*-CAG" (blue), "*Streptococcus*-CAG" (green), "*Lactobacillus*-CAG" (fuchsia), "*Klebsiella*-CAG" (purple).

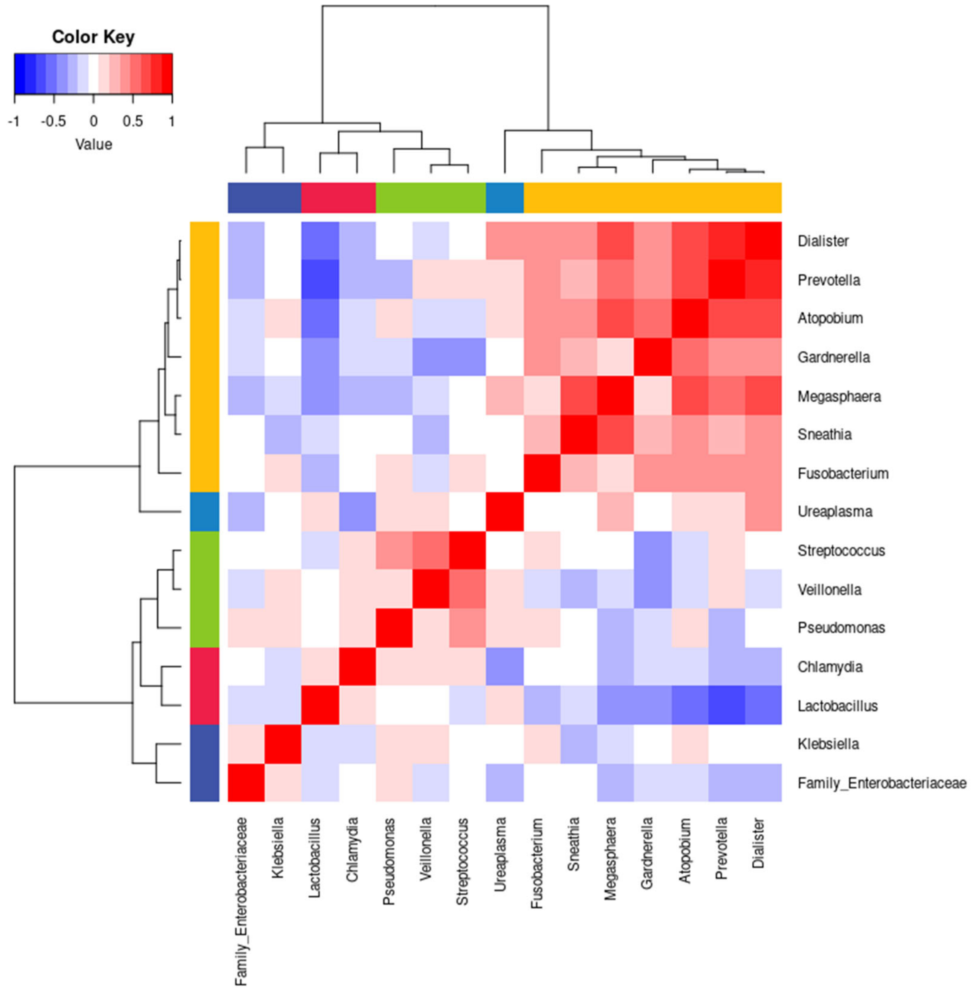

Table S1. Taxonomic relative abundances at phylum, family, genus levels. Average relative abundances and standard deviation values are reported for each infection status (not-infected patient, CT-; CT-infected patient, CT+) at the different phylogenetic levels, along with statistical p-values (non-parametric Mann–Whitney U test); p<0.05 were considered significant.

**Taxonomy**			**Average (SD)**			
**Phylum**	**Family**	**Genus**	**CT-**	**CT+**	***p***	**significance**
Firmicutes			77.17 (19.93)	66.43 (26.36)	0.205	
	Lactobacillaceae		66.52 (28.28)	62.3 (28.92)	0.693	
		Lactobacillus	66.52 (28.28)	62.3 (28.92)	0.693	
	Veillonellaceae		5.08 (13.3)	1.36 (2.57)	0.369	
		Veillonella	3.09 (13.15)	0.03 (0.03)	0.449	
		Dialister	1.53 (3.37)	0.51 (0.73)	0.329	
		Megasphaera	0.42 (1.63)	0.82 (1.89)	0.536	
Actinobacteriota			7.96 (9.99)	10.92 (17.64)	0.547	
	Bifidobacteriaceae		6.83 (9.57)	9.98 (17.12)	0.507	
		Gardnerella	6.38 (9.79)	9.94 (17.12)	0.457	
		Bifidobacterium	0.37 (1.17)	0.01 (0.02)	0.310	
	Atopobiaceae		0.47 (1.02)	0.68 (1.59)	0.659	
		Atopobium	0.47 (1.02)	0.68 (1.59)	0.658	
		Corynebacterium	0.30 (0.59)	0.11 (0.23)	0.313	
Proteobacteria			7.13 (10.4)	12.41 (18.85)	0.312	
	Pseudomonadaceae		4.05 (6.14)	5.98 (7.87)	0.451	
		Pseudomonas	4.05 (6.14)	5.98 (7.87)	0.451	
	Enterobacteriaceae		2.2 (9.15)	6.02 (19.25)	0.449	
		Uncl. Enterobacteriaceae	2.00 (9.18)	0.14 (0.42)	0.508	
-	-	*Klebsiella*	0.02 (0.08)	5.81 (19.28)	0.172	
Bacteroidetes			6.29 (10.21)	6.07 (12.40)	0.957	
	Prevotellaceae		5.74 (9.39)	5.59 (11.34)	0.967	
		Prevotella	5.58 (9.15)	5.58 (11.33)	1	
	Streptococcaceae		0.61 (1.34)	0.35 (0.51)	0.534	
		Streptococcus	0.61 (1.34)	0.35 (0.51)	0.534	
	Porphyromonadaceae		0.34 (1.18)	0.42 (1.04)	0.858	
		Porphyromonas	0.34 (1.18)	0.42 (1.04)	0.852	
		Chlamydia	0.01 (0.03)	2.13 (5.10)	0.063	
Fusobacteriota			1.22 (4.88)	1.92 (4.00)	0.682	
	Leptotrichiaceae		0.72 (3.04)	1.64 (4.07)	0.472	
	Fusobacteriaceae		0.50 (1.84)	0.28 (0.54)	0.707	
		Sneathia	0.72 (3.04)	1.64 (4.07)	0.472	
		Fusobacterium	0.5 (1.84)	0.28 (0.54)	0.707	

Other phyla				
Mycoplasmataceae	3.24 (3.60)	0.37 (0.53)	0.014	*
Ureaplasma	3.11 (3.44)	0.34 (0.53)	0.013	*
